# Interleukin 26 Skews Macrophage Polarization Towards M1 Phenotype by Activating cJUN and the NF-κB Pathway

**DOI:** 10.3390/cells9040938

**Published:** 2020-04-10

**Authors:** Yi-Hsuan Lin, Yi-Hsun Wang, Yi-Jen Peng, Feng-Cheng Liu, Gu-Jiun Lin, Shing-Hwa Huang, Huey-Kang Sytwu, Chia-Pi Cheng

**Affiliations:** 1 Graduate Institute of Life Sciences, National Defense Medical Center, Taipei 114, Taiwan; kinokochiachia@gmail.com (Y.-H.L.); mysoul471bs@gmail.com (Y.-H.W.); 2 Department of Pathology, Tri-Service General Hospital, National Defense Medical Center, Taipei 114, Taiwan; yijen0426@gmail.com; 3 Rheumatology/Immunology and Allergy, Department of Medicine, Tri-Service General Hospital, National Defense Medical Center, Taipei 114, Taiwan; lfc10399@yahoo.com.tw; 4 Department and Graduate Institute of Biology and Anatomy, National Defense Medical Center, Taipei 114, Taiwan; lingujiun@mail.ndmctsgh.edu.tw; 5 Department of General Surgery, En Chu Kong Hospital, New Taipei 237, Taiwan; h610129@gmail.com; 6 Department of General Surgery, Tri-Service General Hospital, National Defense Medical Center, Taipei 114, Taiwan; 7 National Institute of Infectious Diseases and Vaccinology, National Health Research Institutes, Zhunan 350, Miaoli County, Taiwan; sytwu@ndmctsgh.edu.tw; 8 Department of Microbiology and Immunology, National Defense Medical Center, Taipei 115, Taiwan

**Keywords:** rheumatoid arthritis, interleukin-26, macrophage differentiation

## Abstract

Interleukin 26 (IL-26) is a new member of the IL-10 family that is highly expressed in rheumatoid arthritis (RA). However, the functions of IL-26 produced by macrophages in RA have not been elucidated. In the present work, we evaluated the effects and the mechanisms of IL-26 on M1 and M2 macrophage differentiation. Human or mouse macrophage cells were treated with lipopolysaccharides (LPS), interferon gamma (IFNγ), or IL-4 alone or concurrently treated with IL-26 to monitor M1 or M2 macrophage subtypes. The expression level of M1 or M2 macrophage genes was evaluated by reverse transcription polymerase chain reaction (RT-PCR) and enzyme-linked immunosorbent assay (ELISA). The molecular mechanisms of downstream signaling activation during differentiation were investigated by immunoblotting assay. Our results found that IL-26 promoted macrophage cells from CD80^+^ M1 macrophage differentiation, not from the CD206^+^ M2 phenotype. The messenger RNA of M1-type macrophage markers tumor necrosis factor alpha (TNFα) and inducible nitric oxide synthase (iNOS) was up-regulated in the IL-26-treated group. Also, the M1-related proinflammatory cytokines TNFα and IL-6 were induced after IL-26 stimulation. Interestingly, IL-10, a cytokine marker of M2 macrophage, was also elevated after IL-26 stimulation. Moreover, the M1-like macrophage stimulated by IL-26 underwent cJUN, nuclear factor kappa B (NF-κB), and signal transducer and activator of transcription 1 (STAT1) activation. Our findings suggested the role of IL-26 in synovial macrophages of active rheumatoid arthritis and provided a new insight into IL-26 as a candidate therapeutic target in rheumatoid arthritis.

## 1. Introduction

Rheumatoid arthritis (RA) is a chronic autoimmune disease characterized by many kinds of immune cells infiltrated in articular joints [1]. Previous work showed that macrophages were highly activated in the sublining synovium of RA [2]. Moreover, the heterogeneity and imbalanced subtype of macrophages are also critical in the pathogenesis of RA [3]. Recently, IL-26, a new member of the IL-10 family produced by Th17, NK, and macrophage cells was highly expressed in RA [4,5].

IL-26, originally named AK155, was found by infecting human T cells with hybridizing *Herpesvirus saimiri* (HVS) in vitro [6]. The IL-26 gene is located nearby IFN-γ and IL-22 on chromosome 12q14. The IL-26 protein has 47% amino acid similarity to human IL-10, but 24.7% of the amino acids are specific. The receptor of IL-26 is composed of the transmembrane receptors IL-20RA and IL-10RB [7]. A study has shown that IL-26 receptors IL-20RA and IL-10RB are particularly common on epidermal cells [8]. The intracellular signaling is through the STAT1, STAT3, ERK, JNK, and AKT signaling pathways [9].

A recent study revealed that IL-26 is abundantly expressed on synovial cells of patients with rheumatoid arthritis, especially on CD68 macrophages, and in the chemotaxis of Th17 [5]. Genetic variation of IL-26 also affects the susceptibility of women with rheumatoid arthritis [10]. However, our previous work found that IL-26 suppresses macrophages from osteoclastogenesis [11]. Moreover, the effects of IL-26 on macrophage subtype differentiation and activation in RA are still unknown. Therefore, clarifying the functions of IL-26 cytokines in macrophage differentiation and activation is critical for understanding the pathogenesis of RA.

## 2. Materials and Methods

### 2.1. Cell Line and Reagents

The murine and human monocyte/macrophage cell lines RAW 264.7 and THP-1 were obtained from the Food Industry Research and Development Institute (Taiwan). Human IL-26 recombinant protein was obtained from MyBiosource (San Diego, USA). Recombinant IL-4, IFNγ, and M-CSF of human or mouse species were obtained from Peprotech (London, UK). Phosphorylated and non-phosphorylated STAT1, STAT3, STAT6, and NF-κB were purchased from Cell Signaling Technology (Danvers, MA, USA). Total cJUN was purchased from Bethyl Laboratories (Montgomery, TX, USA). Phosphorylated cJUN was purchased from Santa Cruz Biotechnology (Santa Cruz, CA, USA). Anti-TATA-box-binding protein (anti-TBP) was purchased from Millipore (Carrigtwohill, Co. Cork, Ireland). Anti-glyceraldehyde 3-phosphate dehydrogenase (GAPDH) was obtained from Proteintech (Chicago, IL, USA). The ELISA kits for IL-6, IL-10, and TNFα were purchased from BioLegend (San Diego, CA, USA), and the ELISA kit for TGFβ was purchased from Invitrogen (Carlsbad, CA, USA). NF-κB inhibitor sn50 and all other reagents were obtained from Sigma Chemical Co. (St. Louis, MO, USA).

### 2.2. M1 M2 Macrophage Differentiation and Inhibition Assay

RAW264.7 cells were seeded at 1 × 10^5^ cells/well in a 24-well plate and treated with or without IL-26 (60 ng/mL) in the presence or absence of LPS (10 ng/mL), IFN-γ (20 ng/mL), or IL-4 (20 ng/mL) for 24 h. Primary murine macrophage bone marrow-derived macrophage (BMDM) was isolated from mice tibia and femur. For macrophage differentiation, BMDMs were treated with M-CSF (50 ng/mL) for 7 days and were then treated with or without IL-26 (60 ng/mL) in the presence or absence of LPS (10 ng/mL), IFN-γ (20 ng/mL), or IL-4 (20 ng/mL) for 24 h. THP-1 cells were pre-treated with PMA for 24 h, allowed to stand for 24 h, and then treated with or without IL-26 (60 ng/mL) in the presence or absence of LPS (10 ng/mL), IFN-γ (20 ng/mL), or IL-4 (20 ng/mL) for 24 h. For AP-1, STAT1, and NF-κB inhibition, RAW264.7 was pretreated with sn50 (50 μg/mL or 75 μg/mL) for 1 h and then treated with a half-dosage of LPS (5ng/mL) or IL-26 (30 ng/mL) for 16 h.

### 2.3. Quantitative RT-PCR Analysis

In brief, RNA was extracted using the NucleoSpin^®^ RNA kit (Macherey-Nagel GmbH & Co. KG, Germany), and SuperScript III reverse transcriptase was used for reverse transcription of RNA to cDNA (Invitrogen). All differentiation markers were analyzed on a LightCycler 480 II system (Roche, Mannheim, Germany). The specific primers are shown in Table S1. The quantitative thermal cycling parameters were 95 ℃ for 15 min, followed by 40 cycles for 30 s at 95 ℃, 30 s at 60 ℃, and 1 min at 72 ℃, followed by extension for 10 min at 72 ℃. The relative levels of each value were evaluated and normalized with GAPDH.

### 2.4. Immunoblotting Analysis

Cytoplasmic and nuclear extracts were separated according to our previous work [11]. In brief, the cytoplasmic fraction was extracted using buffer A followed by centrifugation for 5 min at 14,000 rpm to obtain the supernatant. The pellet was resuspended in buffer C, incubated on ice, and centrifuged for 5 min at 14,000 rpm at 4 °C as the nuclear fraction. Equal amounts of total protein were used for immunoblotting by using specific antibodies, cJUN, phospho-cJUN, NF-κB p65, phospho-NF-κB p65, TBP, IRF5, STAT1, phospho-STAT1, STAT3, phospho-STAT3, STAT6, phospho-STAT6, and GAPDH. 

### 2.5. Flow Cytometry

After polarization of each group, cells were harvested and stained with M1 anti-CD80 FITC and anti-CD86PE or M2 anti-CD206 PE cell surface maker (BD Biosciences, NJ, USA). After 30 min incubation, cells were washed and then analyzed by a FACSCalibur flow cytometer and CellQuest Pro software (BD Biosciences, NJ, USA).

### 2.6. ELISA

After polarization, cell media were harvested for further ELISA analysis. All procedures were according to the manufacturer. In brief, the following steps were performed: precoat the IL-6, IL-10, TNFα, or TGFβ captured antibodies to all wells of a 96-well plate and incubate overnight between 2 °C and 8 °C. Wash the plate and then incubate it with blocking buffer for 1 h. Add 100 μL/well of standards or samples and incubate at room temperature for 2 h with shaking. Wash the plate and then incubate it with IL-6, IL-10, TNFα, or TGFβ detection antibody for 1 h with shaking. Wash the plate and then incubate it with Avidin-HRP solution for 30 min with shaking. Wash the plate and then incubate it with TMB substrate solution for 20 min in the dark. Finally, add stop solution to each well and read the absorbance at 450 or 570 nm within 15 min.

### 2.7. MTT Assay

Cells at a density of 5 × 10^4^ cells/mL were cultured in a 96-well plate and treated with different concentrations of IL-26 for 24 and 48 h. After 24 or 48 h, cells were harvested and washed with phosphate-buffered saline (PBS) three times following by culturing with medium containing 500 µg/mL of 3-(4,5-dimethylthiazol-2-yl)-2,5-diphenyl- tetrazolium bromide (MTT) for 30 min at 37 ℃. Then, cells were harvested and dissolved in 100 µl of DMSO. The concentrations of purple formazan from the intracellular solution were determined by an ELISA reader at 550 nm.

### 2.8. Statistical Analysis

Data are presented as means ± SD and were analyzed by using one-way ANOVA with Newman–Keuls multiple comparisons posttests. *p* < 0.05 was considered statistically significant.

## 3. Results

### 3.1. Effects of IL-26 on Macrophage Differentiation

**Figure 1:**
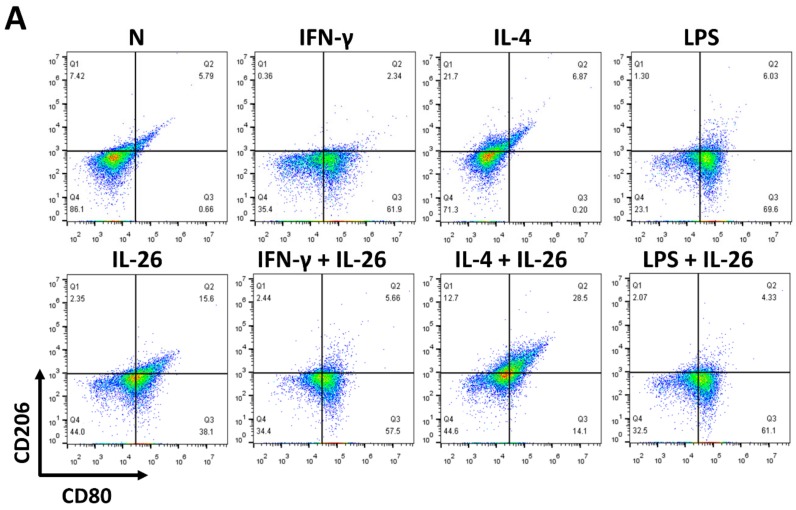

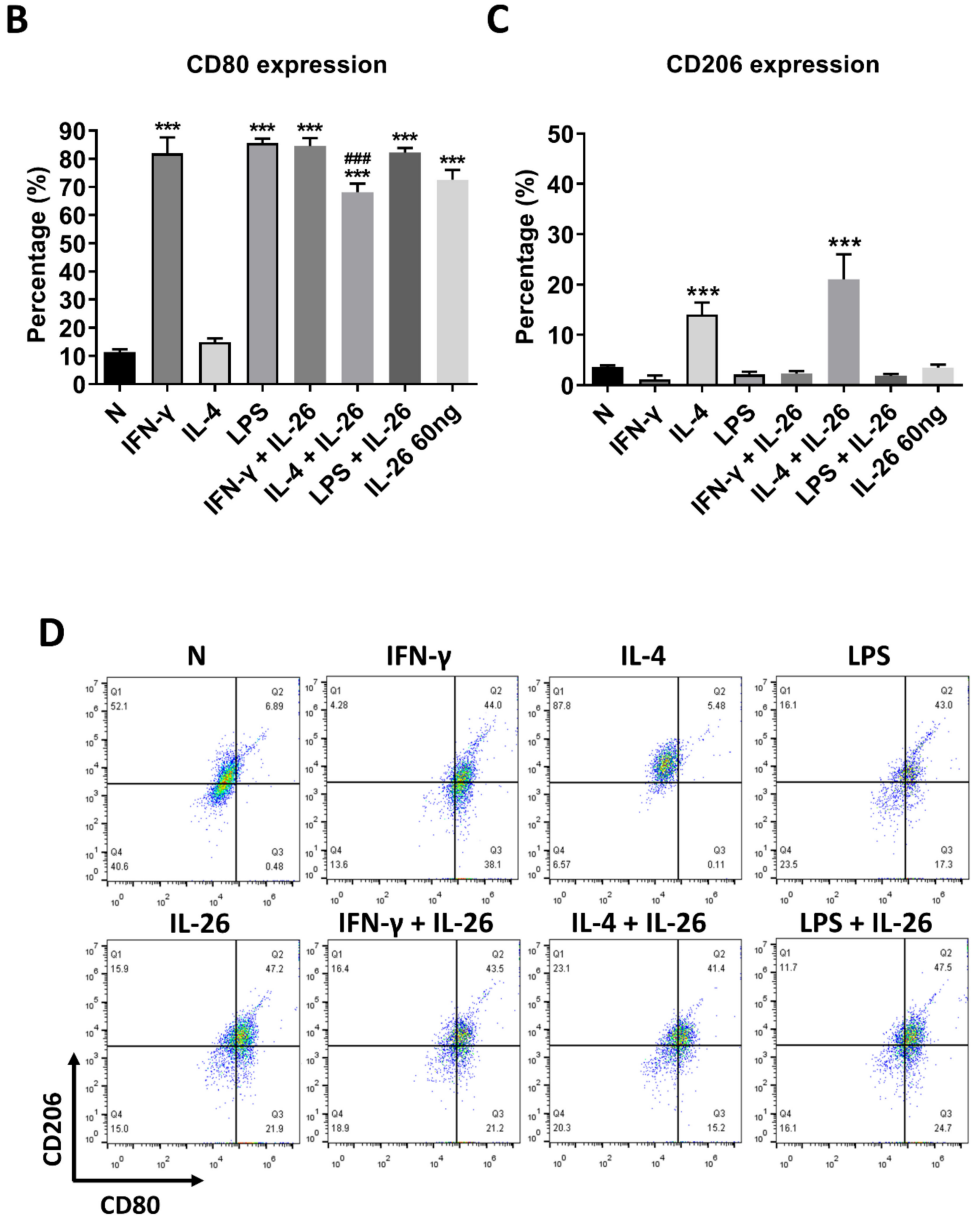

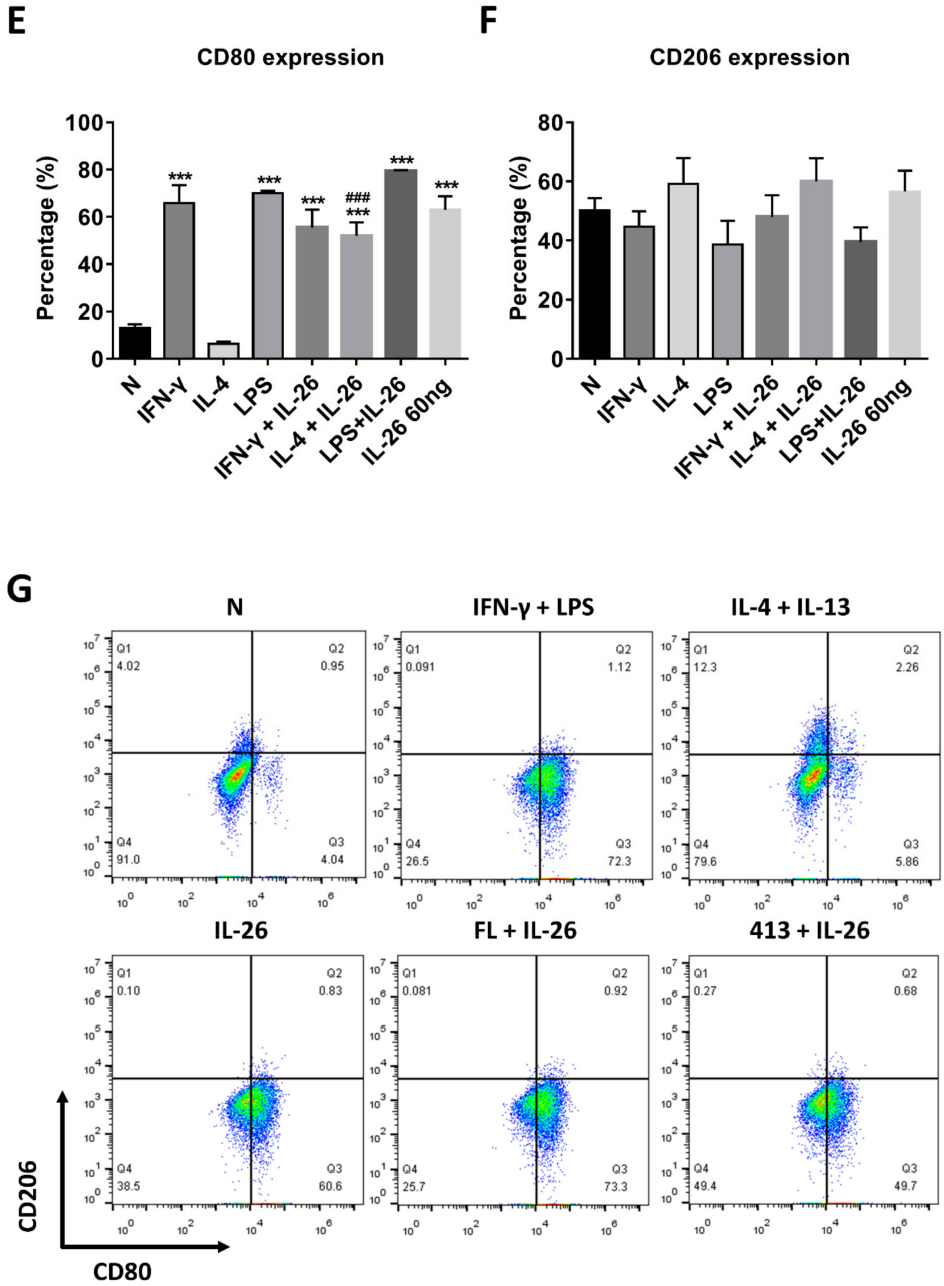

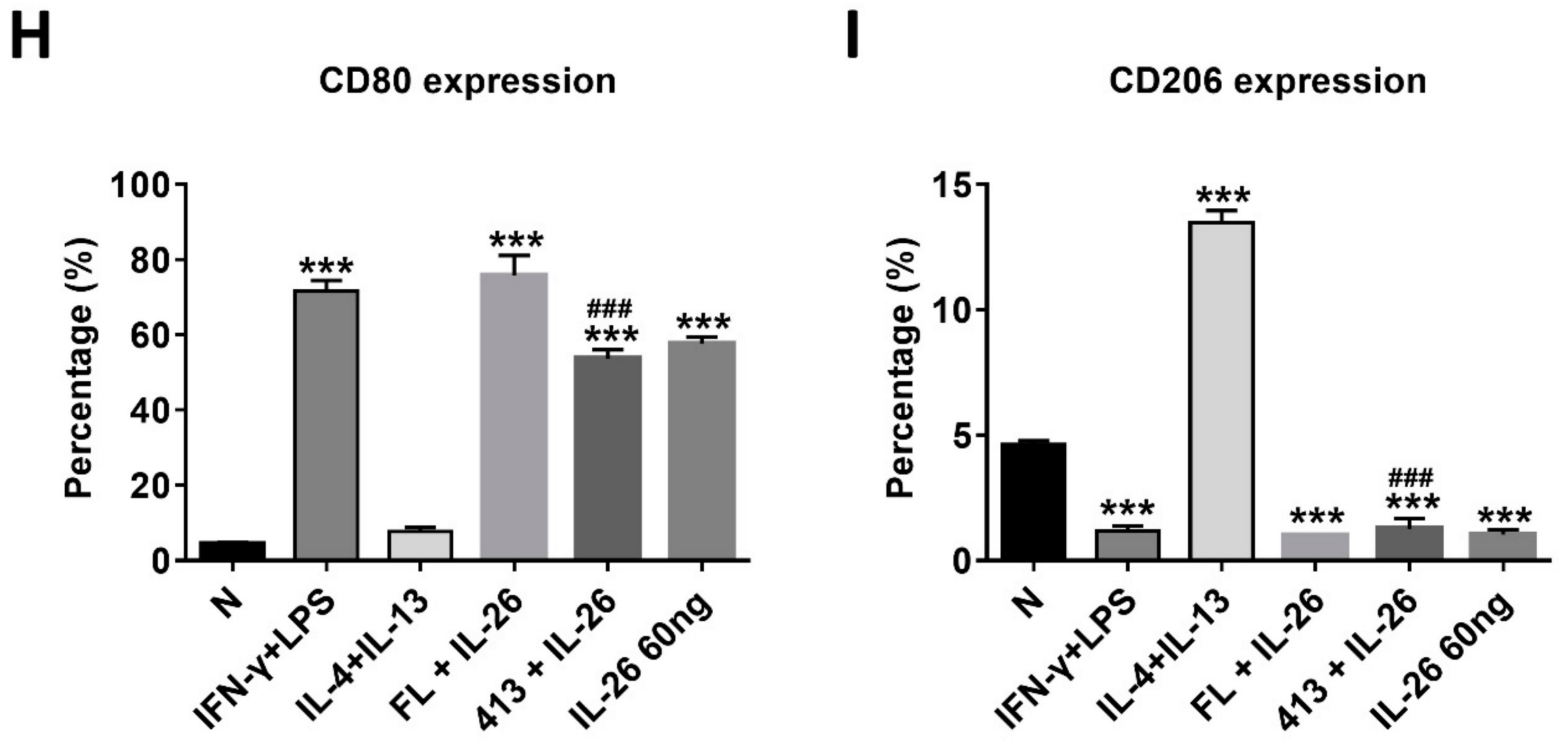
Effect of IL-26 on M1 and M2 macrophage differentiation in RAW264.7, BMDM, and THP-1. (**A**–**C**) RAW264.7 cells were treated with IFN-γ (20 ng/mL), IL-4 (20 ng/mL), or LPS (10 ng/mL) in the presence or absence of IL-26 for 24 h. (**D**–**F**) BMDM cells were pretreated with M-CSF (50 ng/mL) for 7 days and then treated with IFN-γ (20 ng/mL), IL-4 (20 ng/mL), or LPS (10 ng/mL) in the presence or absence of IL-26 for 24 h. (**G**–**I**) THP-1 cells treated with IFN-γ (20 ng/mL) plus LPS (10 ng/mL) or IL-4 (20 ng/mL) plus IL-13 (20 ng/mL) in the presence or absence of IL-26 for 24 h. After incubation, the cells were stained with CD80 and CD206 to separate CD80 M1 and CD206 M2 macrophages. Results are the means ± SD of three independent experiments. (*** *p* < 0.001, ^###^ *p* < 0.001 multiple comparison significance between IL-4 and IL-4+IL-26 or IL-4+IL-13 and IL-4+IL-13+IL-26).

At the beginning of the experiments, we treated cells with various concentrations of IL-26 for 24 and 48 h for measuring cell viability by MTT assay. The result shows that the safety dosage of IL-26 in RAW264.7, BMDM, and THP-1 was below 120 ng/mL (Figure S1). To examine the effects of IL-26 on macrophage differentiation, we treated RAW264.7 cells in the presence or absence of IL-26 concurrent with traditional M1 and M2 macrophage-differentiated cytokine followed by flowcytometry analyzing with CD80, CD86, and CD206 surface markers. Our data show that IL-26 dominantly promoted macrophage cells toward CD80^+^ or CD86^+^ (Figure S2) not CD206^+^ cell differentiation on RAW264.7 cells (Figure 1A–C). Interestingly, our previous study found that IL-26 suppressed osteoclast formation. In this regard, the result implied that IL-26 promotes monocytes with a potential tendency to differentiate into M1 macrophages. To test the hypothesis, we treated BMDM cells with MCSF to differentiate the cells toward the M2 phenotype, and then combined treatment with IL-26. The result shows that the differentiation of BMDM cells was switched from CD206^+^ CD80^-^ M2 toward the CD206^+^ CD80^+^ M1 phenotype after IL-26 addition (Figure 1D-F). Moreover, in order to check the phenomenon was a cross-species event between mouse and human, we further analyzed the human macrophage cell line, THP-1, by polarizing it with or without IL-26 in the presence or absence of M1 or M2 macrophage-differentiated cytokines. The result showed that human THP-1 cells were similar to mouse cells with a tendency of CD80^+^ M1 (Figure 1G–I).

### 3.2. Effects of IL-26 on the Gene Expression of M1 and M2 Macrophage Differentiation

**Figure 2:**
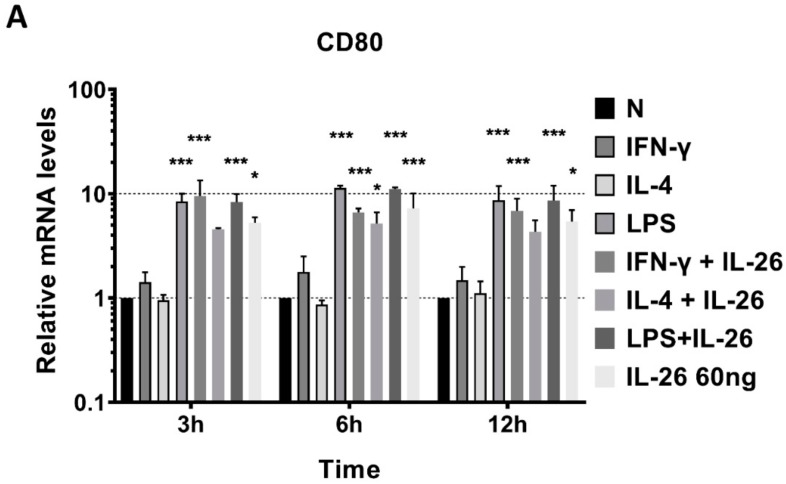

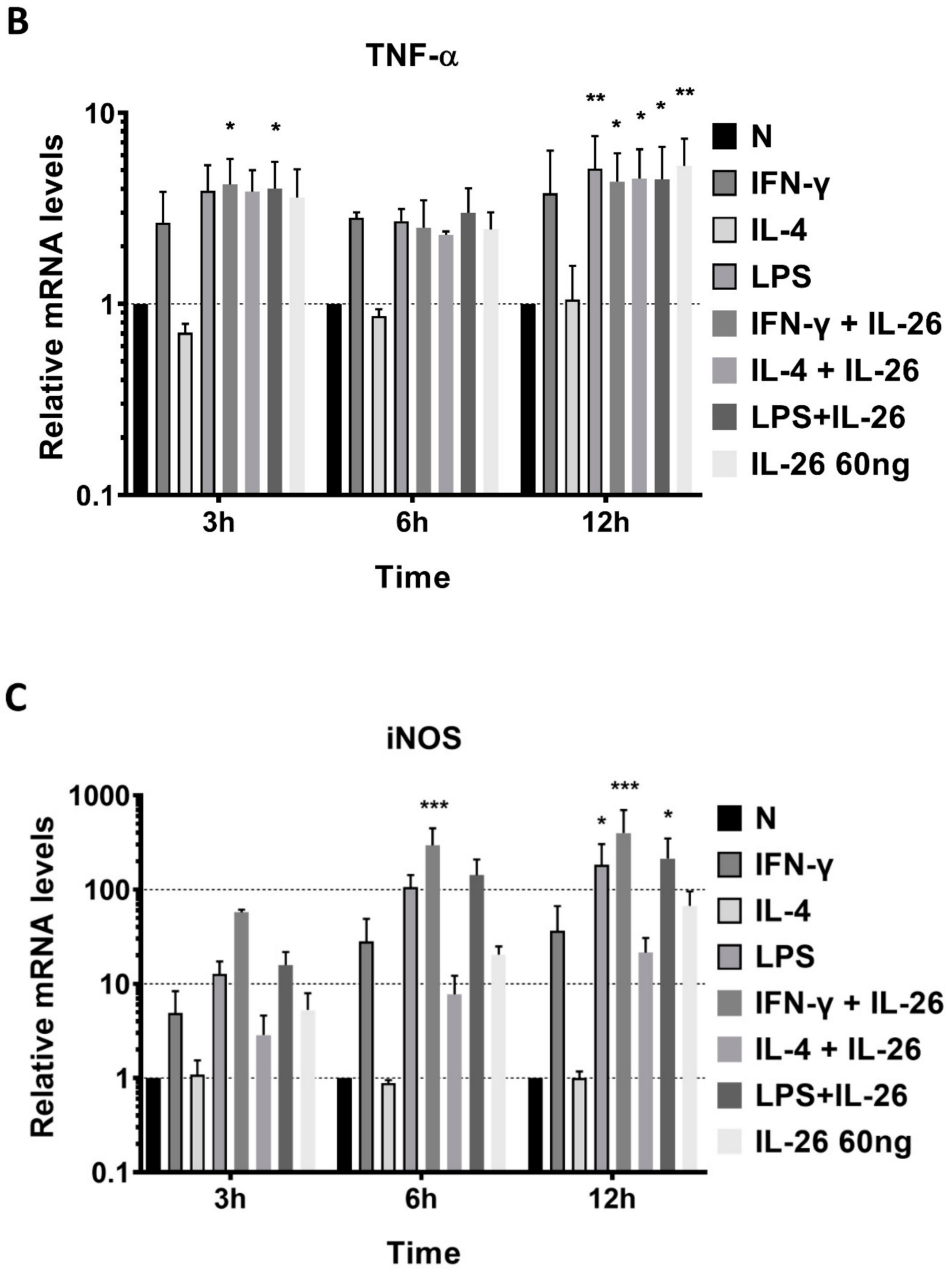

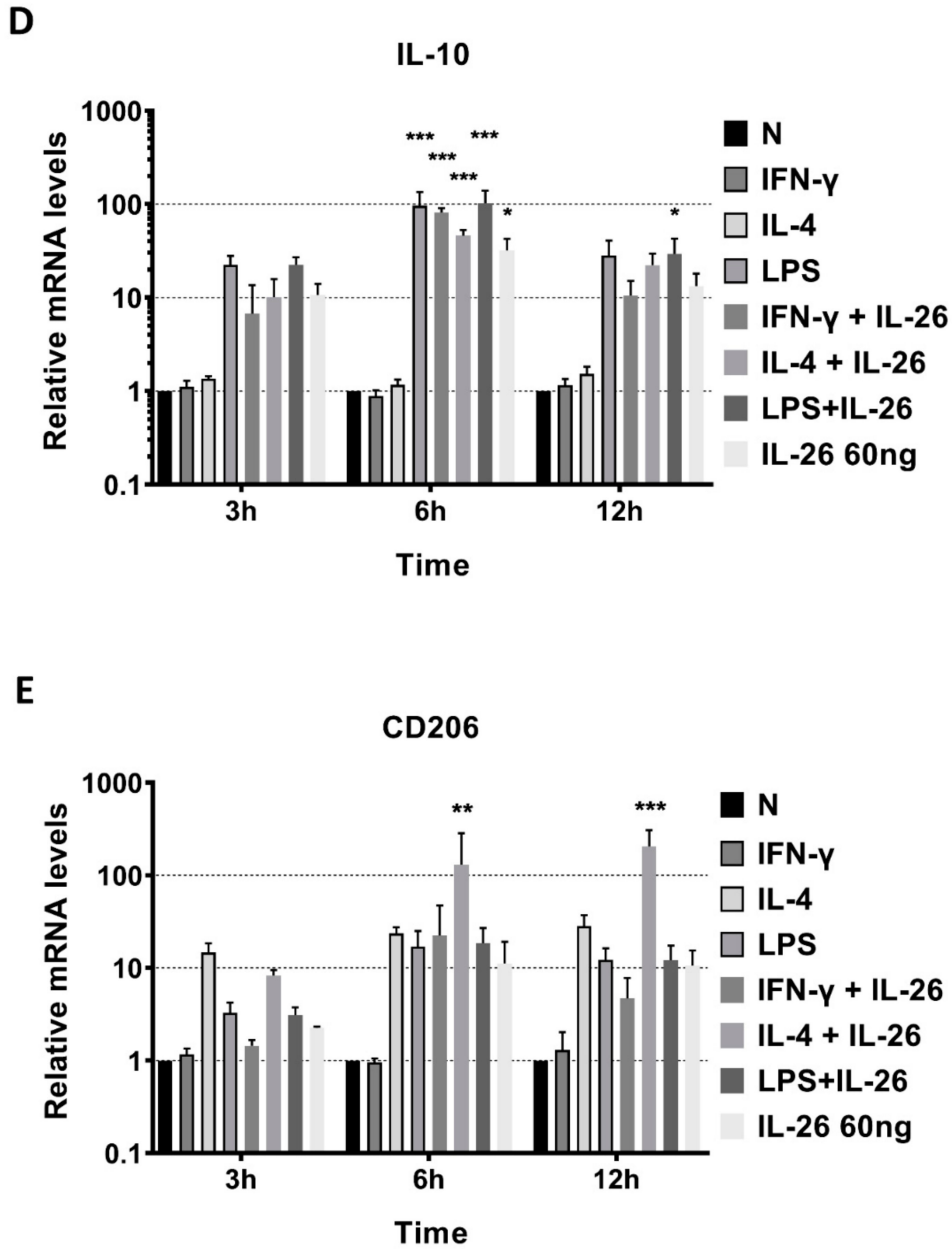
Effects of IL-26 on mRNA expression in M1 and M2 macrophage differentiation. RAW264.7 cells were treated with IFN-γ (20 ng/mL), IL-4 (20 ng/mL), or LPS (10 ng/mL) in the presence or absence of IL-26 for 3, 6, and 12 h. Total RNA was isolated, and 1 μg of total RNA was used to transcribe cDNA. cDNA was used as a template for PCR with mouse-specific primers. M1 macrophage gene markers (**A**) CD80, (**B**) TNF-α, or (**C**) iNOS, and M2 macrophage gene markers (**D**) IL-10 or (**E**) CD206 were detected by QPCR. Results are the means ± SD of three independent experiments. (* *p* < 0.05, ** *p* < 0.01, *** *p* < 0.001).

To further examine the phenotype of IL-26 in macrophage differentiation, we analyzed the mRNA level of traditional M1, CD80, inducible nitric oxide synthase (iNOS), and TNFα genes and the M2, IL-10, and CD206 gene expression by QPCR. The supernatant of secretory M1 cytokines, IL-6 and TNFα, and M2 cytokines, IL-10 and TGFβ, was detected by ELISA. Results revealed that inducible M1 macrophage mRNA, CD80, iNOS, and TNFα were significantly increased in RAW264.7 cells after IL-26 stimulation (Figure 2).

**Figure 3:**
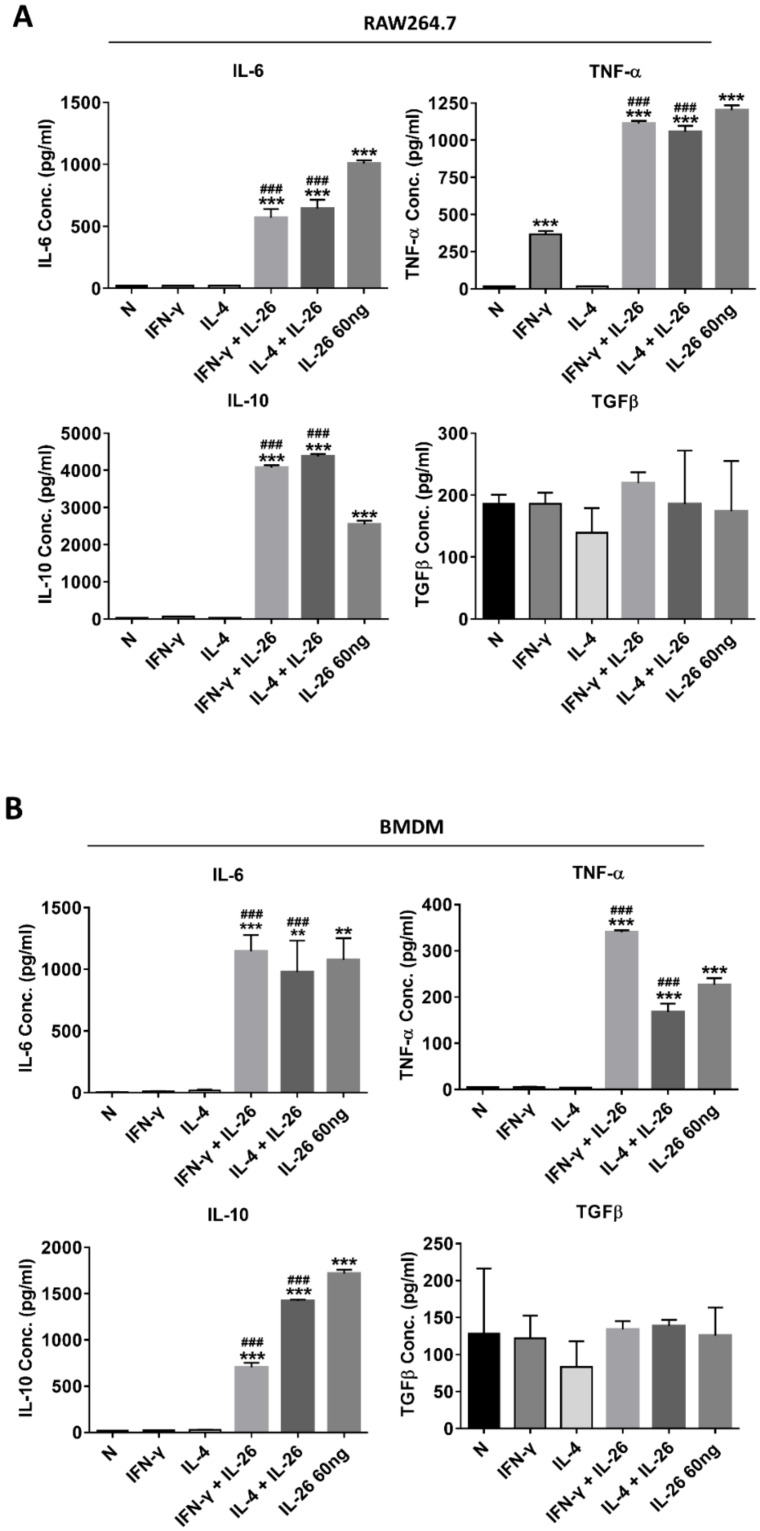

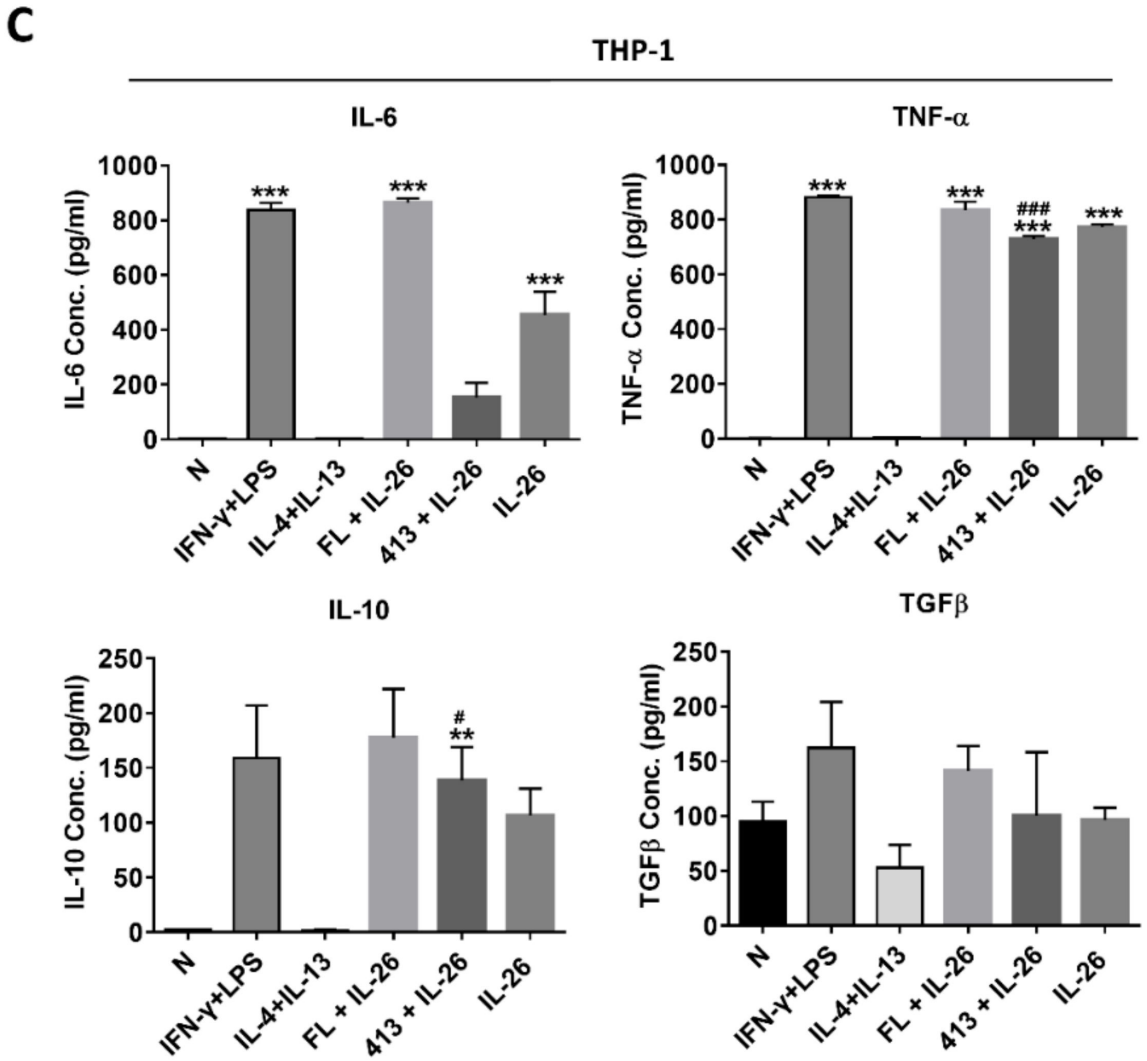
Effect of IL-26 on cytokine expression in M1 and M2 macrophage differentiation. (**A**) RAW264.7 cells were treated with IFN-γ (20 ng/mL) or IL-4 (20 ng/mL) in the presence or absence of IL-26 for 24 h. (**B**) BMDM cells were pretreated with M-CSF (50 ng/mL) for 7 days and then treated with IFN-γ (20 ng/mL) or IL-4 (20 ng/mL) in the presence or absence of IL-26 for 24 h. (**C**) THP-1 cells treated with IFN-γ (20 ng/mL) plus LPS (10 ng/mL) or IL-4 (20 ng/mL) plus IL-13 (20 ng/mL) in the presence or absence of IL-26 for 24 h. After incubation, the cultured supernatants were analyzed by M1 macrophage cytokines IL-6 and TNF-α and M2 macrophage cytokines IL-10 and TGFβ by ELISA. The data are the means ± S.D. of more than three cultures (* *p* < 0.05, ** *p* < 0.01, *** *p* < 0.001; ^#^ *p* <0.05, ^###^ *p* < 0.001 multiple comparison significance between IL-4 and IL-4+IL-26, IFNγ and IFNγ+IL-26, or IL-4+IL-13 and IL-4+IL-13+IL-26 ).

Also, the secretory proteins of M1 cytokines, IL-6, and TNFα were both highly expressed in IL-26 treated alone or combined with the M1 or M2 differentiation-treated group in RAW264.7, BMDM, and THP-1 (Figure 3). Interestingly, the M2 cytokine, IL-10, was also elevated in IL-26 alone or combined with the IFNγ or IL-4 treated group.

### 3.3. Effects of IL-26 on STAT Activation of M1 and M2 Macrophage Differentiation

**Figure 4:**
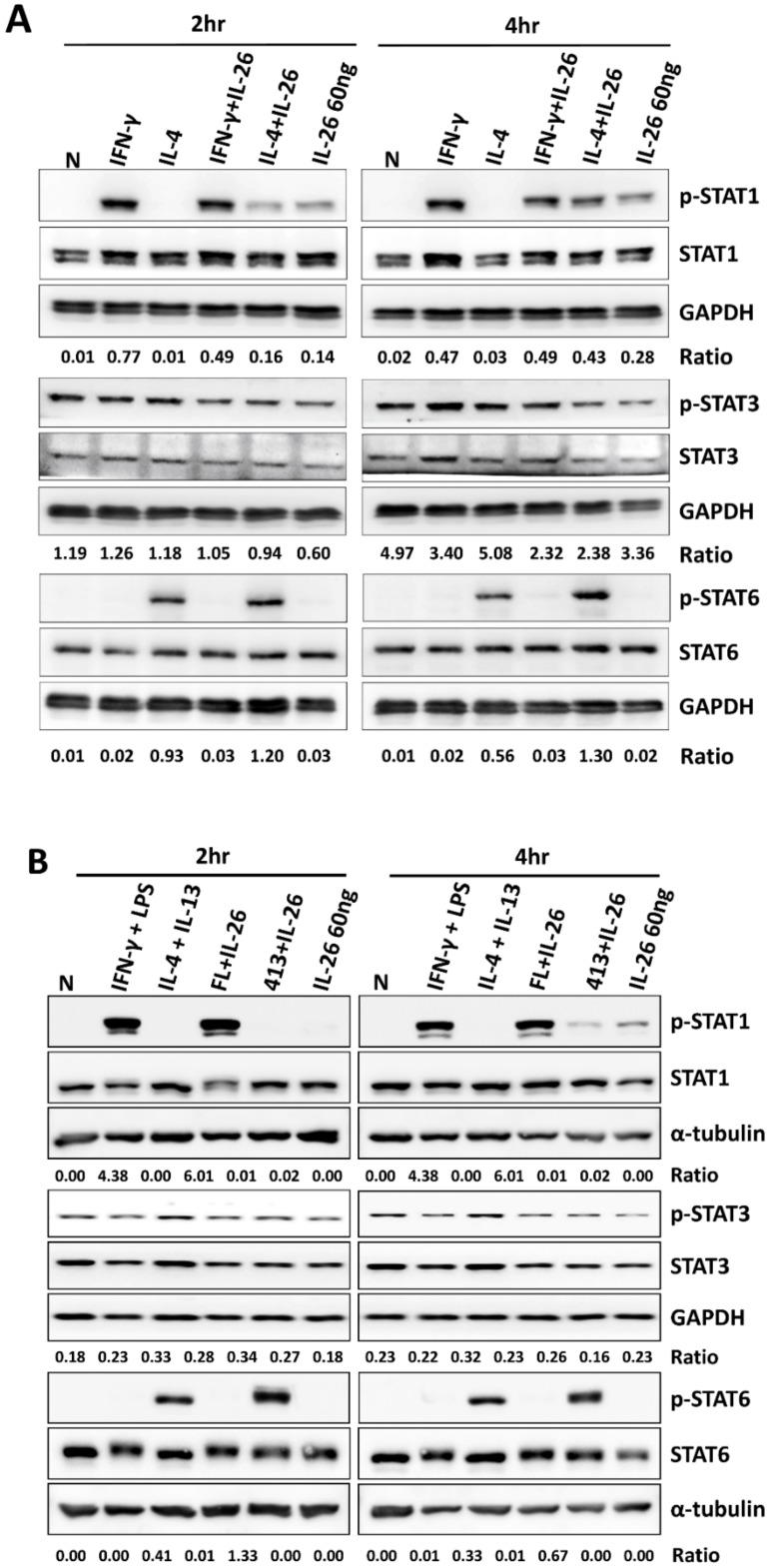
Effect of IL-26 on STAT1, STAT3, and STAT6 activation in M1 and M2 macrophage differentiation. (**A**) RAW264.7 cells were serum-starved for 16 h and treated with IFN-γ (20 ng/mL) or IL-4 (20 ng/mL) in the presence or absence of IL-26, and (**B**) THP-1 cells were treated with IFN-γ (20 ng/mL) plus LPS (10 ng/mL) or IL-4 (20 ng/mL) plus IL-13 (20 ng/mL) in the presence or absence of IL-26 for 2 and 4 h to further detect phosphorylated or non-phosphorylated STAT1, STAT3, and STAT6 proteins. Cell extracts were analyzed by western blot using antibodies specifically directed against the phosphorylated forms of STATs, compared with data obtained with antibodies directed against the unphosphorylated states of the kinases. Equal amounts of protein were loaded in each lane as demonstrated by the level of GAPDH. A representative result of at least three independent experiments is shown. (FL+IL-26: IFN-γ+LPS+IL-26; 413+IL-26: IL-4 + IL-13+IL-26)

A previous study reported that STAT1 and STAT3 were activated by IL-26 in colon cancer carcinoma [8]. Also, STAT1 has been reported to play a pivotal role in M1 macrophage differentiation; however, STAT3 and STAT6 activation was dominant in M2 macrophage. Therefore, we examined the effects of IL-26 on STAT1, STAT3, and STAT6 activation during M1 and M2 macrophage polarization in a dose- and time-dependent manner. Our results revealed that only STAT1 was activated by IL-26 stimulation at 120 min in M1 and M2 macrophage differentiation even if combined with IL-4 in both RAW264.7 and THP-1 cells (Figure 4, the quantified and statistical analysis is shown in Figure S3).

### 3.4. Effects of IL-26 on Nuclear Translocation and Activation of IRF5, cJUN, and NF-κB during M1 and M2 Macrophage Differentiation

**Figure 5:**
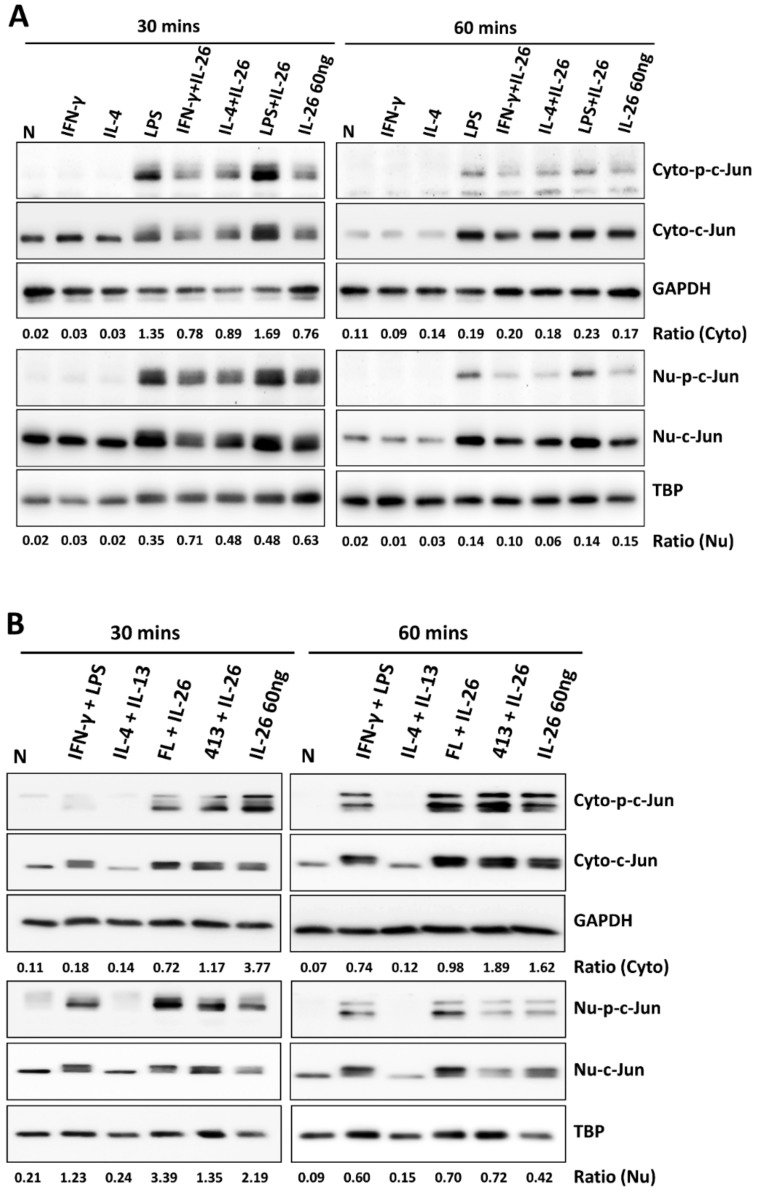

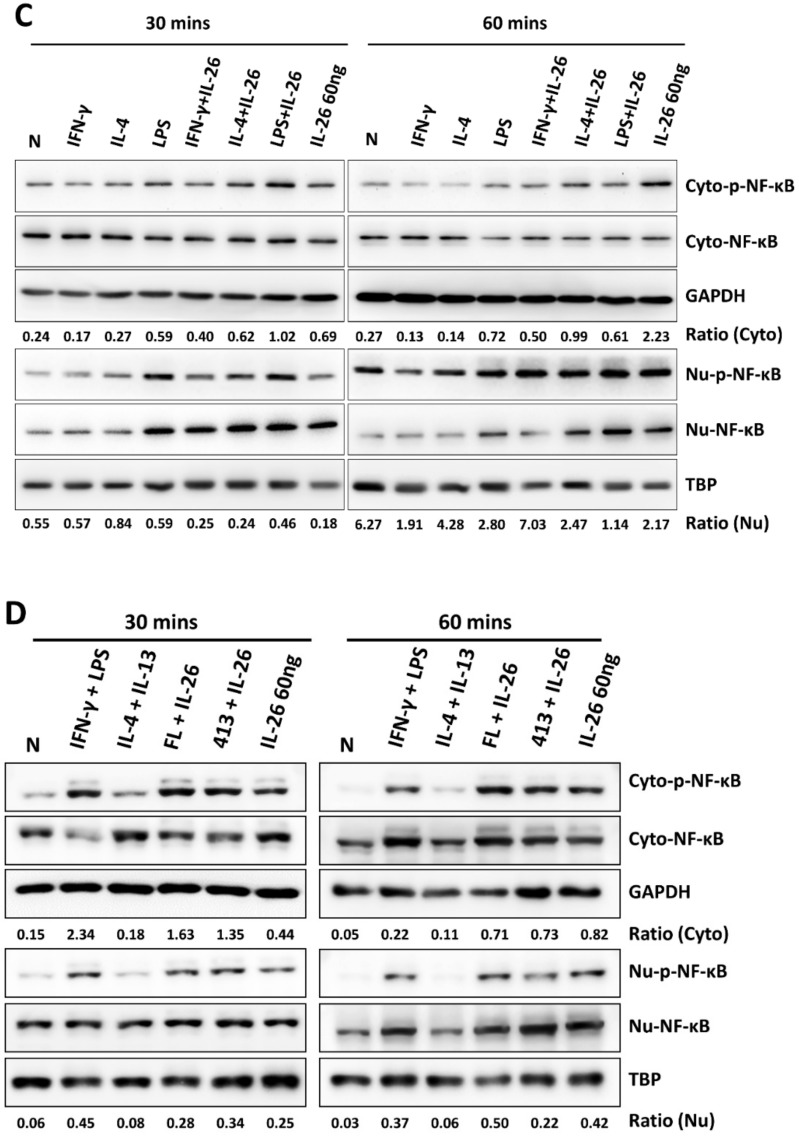
Effect of IL-26 on cJUN and NF-κB activation in M1 and M2 macrophage differentiation. (**A**,**C**) RAW264.7 cells were serum-starved for 16 h and then treated with IFN-γ (20 ng/mL), IL-4 (20 ng/mL), or LPS (10 ng/mL) in the presence or absence of IL-26 for 30 or 60 min. (**B**,**D**) THP-1 cells were treated with IFN-γ (20 ng/mL) plus LPS (10 ng/mL) or IL-4 (20 ng/mL) plus IL-13 (20 ng/mL) in the presence or absence of IL-26 for 30 or 60 min. Cytoplasmic extracts (Cyto) and nuclear extracts (Nu) were analyzed by western blot using an antibody specifically directed against phosphorylated or non-phosphorylated c-JUN (**A**,**B**) and NF-κB (**C**,**D**) proteins. Equal amounts of protein were loaded in each lane as demonstrated by the level of TBP, nuclear internal control, and GAPDH, cytoplasmic internal control. A representative result of at least three independent experiments is shown. (FL+IL-26: IFN-γ+LPS+IL-26; 413+IL-26: IL-4 + IL-13+IL-26).

During M1 macrophage differentiation, activation of NF-κB is critical in inflammatory gene expression and is synergized with STAT1 activation [12]. A previous study also reported that IRF5 was involved in regulating M1 macrophage polarization and gene expression [13]. Moreover, c-JUN has been reported as a key subunit of the AP-1 transcription factor which is involved in NF-κB activation and arthritis development [14]. To further investigate which transcription factor was the guide of IL-26 in M1 macrophage differentiation transcriptional regulation, we examined the effect of IL-26 on M1 and M2 macrophage polarization by detecting transcription factors IRF5, cJUN, and NF-κB activation and nuclear translocation. Our results found that IL-26 enhanced cJUN and NF-κB phosphorylation, in both the cytoplasm and nucleus (Figure 5, the quantified and statistical analyses are shown in Figure S4). However, the result shows that IRF5 was not affected by IL-26 during nuclear translocation (Figure S5). Furthermore, inhibition of AP-1, STAT1, and NF-κB activation by the sn50 inhibitor would restrict the dominant CD80 M1 macrophage expression (Figure S6).

## 4. Discussion

In this study, IL-26 directly promoted macrophage cells toward M1 phenotype differentiation and switched M-CSF-treated M2 macrophage cells to the M1 phenotype. In the past decade, most studies focused on the function of IL-26 working in Th17 or NK cells. For instance, *Manel* et al. found that during Th17 cell differentiation, IL-17F, the IL-23 receptor, CCR6, and IL-26 gene expression could be induced by RORγt [15]. NK-22, a human NK cell subset, secretes leukemia inhibitory factors (LIFs), IL-22, and IL-26 [16]. Also, NK cells overexpressed IL-26 in HCV-infected patients [17]. Moreover, previous studies reported that IL-26 is involved in skin and mucosa immunity. IL-26 was expressed in psoriasis patients in combination with the IL-26 receptor, IL-10RB, and IL-20RA expressed by keratinocytes [18,19]. IL-26 stimulates neutrophils and accumulates immune cells against bacteria in human lung infections [20]. Recently, IL-26 was regarded as a critical cytokine for extracellular DNA-induced inflammation and bacterial infection [21,22]. IL-26 can be rapidly induced by IL-1β in Th17 Cells [23]. All evidence shows that IL-26 is a response element of foreign pathogens and functions in regulating immunity. Our previous work found that IL-26 could directly inhibit macrophages from osteoclast differentiation. Here, we found that IL-26 not only inhibits osteoclastogenesis but also directly affects macrophage subtype differentiation.

The pathogenic phenotype of the macrophages in RA were heterogenous and still need to be elucidated. A previous study reported that macrophages were the most abundant cell type in a synovial biopsy of RA [24]. The cytokine profile in the serum and synovial fluid revealed dominant expression of TNFα, IL-1, and IL-6 in RA [25]. Granulocyte–macrophage colony-stimulating factor (GM-CSF) is activated by TNFα and IL-1 in synovial cells which promotes monocyte/macrophage cell differentiation and induces HLA class II expression [26]. Thus, active RA is regarded as an M1 macrophage-dominant disease based on these cytokines and growth factor. Furthermore, many M1 macrophage transcription factors are involved in the disease process, such as NF-κB, AP-1, and IRF5. NF-κB has been reported as a pivotal transcription factor during inflammation in synovial T cells, macrophages, and fibroblasts of RA [27,28]. A recent study reported that the subunit of AP-1 transcription factor c-Jun promotes arthritis in macrophages by regulating cyclooxygenase-2 and arginase-1 expression [14]. Genetic deficiency of IRF5 in an arthritis animal model could reduce arthritis severity [29]. In our study, we found that IL-26 markedly activated NF-κB and cJUN phosphorylation to promote M1 macrophage differentiation. Moreover, the M1 macrophage-related downstream genes, iNOS and TNFα, were upregulated. Also, the M1 representative proinflammatory cytokines, IL-6 and TNFα, were also enhanced after IL-26 stimulation.

Based on IL-26 specific signaling transduction, previous studies reported that the receptor of IL-26 was composed of a heterodimeric receptor consisting of IL-10RB and IL-20RA, and the intracellular molecular mechanisms were transduced by the STAT1 and STAT3 signaling pathways [7,8]. A previous study reported that both STAT1 and STAT3 were key transcription factors for M1 macrophage differentiation [30]. However, the receptor of the IL-20RA subunit is not expressed on monocytes and macrophages [31]. Activation of STAT3 in HT-29 colorectal carcinoma by IL-26 was dependent on the expression of IL-20RA [32]. Therefore, we could presume that STAT3 is not one of the major molecular mechanisms involved in IL-26-induced M1 macrophage differentiation. Our hypothesis was consistent with our findings, we discovered that only STAT1 phosphorylation was transduced in M1 macrophage differentiation after IL-26 stimulation, and STAT3 was not affected. Moreover, a previous study reported that anti-inflammatory treatments of RA would increase the infection risk of tuberculosis [33]. Activation of STAT1 and c-JUN is a possible mechanism in macrophages for patients to survive against active tuberculosis [34]. Also, c-JUN has been proved to engage in functional crosstalk between interferon signaling pathway by regulating the expression of STAT1 [35]. Our findings indicate that IL-26-stimulated macrophages toward the M1 phenotype would activate c-JUN and NF-κB transcription factors immediately and then subsequently activate STAT1 signaling in consistent with the pathophysiology of RA. Interestingly, IL-10, a M2 dominantly expressed cytokine, was also elevated after IL-26 stimulation. This might be due to the homology between IL-26 and IL-10. The underlying molecular mechanisms need to be further investigated.

In conclusion, we provided new evidence that IL-26 could directly promote monocyte/macrophage cells during M1 macrophage differentiation. We also provided data related to phenotypic expression as well as M1 macrophage-related gene regulation of IL-26-stimulated macrophage differentiation. Our findings indicate that IL-26-stimulated macrophages are involved in the molecular mechanisms of the regulation of the STAT1, c-JUN, and NF-κB pathways. Finally, IL-26-stimulated M1 macrophages enhanced the expression of proinflammatory cytokines TNF-α and IL-6 to regulate the immune system. Taken together, our findings show the molecular mechanisms of IL-26 involved in macrophage subtype regulation of the innate immune system and might make IL-26 a potential therapeutic target for M1-dominant autoimmune or inflammatory diseases such as RA.

## Supplementary Materials

The following are available online at https://www.mdpi.com/2073-4409/9/4/938/s1, Figure S1. Effects of IL-26 on viability of RAW264.7, BMDM, and THP-1; Figure S2. Effect of IL-26 on M1 CD80/CD86 macrophage marker expression in RAW264.7; Figure S3. Effect of IL-26 on STAT1, STAT3, and STAT6 activation in M1/M2 macrophage differentiation; Figure S4. Effect of IL-26 on cJUN and NF-κB activation in M1/M2 macrophage differentiation; Figure S5. Effect of IL-26 on IRF5 translocation in M1/M2 macrophage differentiation; Figure S6. Suppression of IL-26 on STAT1, AP-1 and NF-κB activation by sn50 inhibitor; Table S1. List of murine PCR primers.
