# Supplementary material for: Interleukin 26 Skews Macrophage Polarization Towards M1 Phenotype by Activating cJUN and the NF-κB Pathway
Source: Cells. 2020 Apr 10;9(4):938. doi: 10.3390/cells9040938 (PMC7227026; doi:10.3390/cells9040938)
Supplement: Supplementary file 1 [file cells-09-00938-s001.pdf]

Supplementary Information

Supplementary Figures

Figure S1

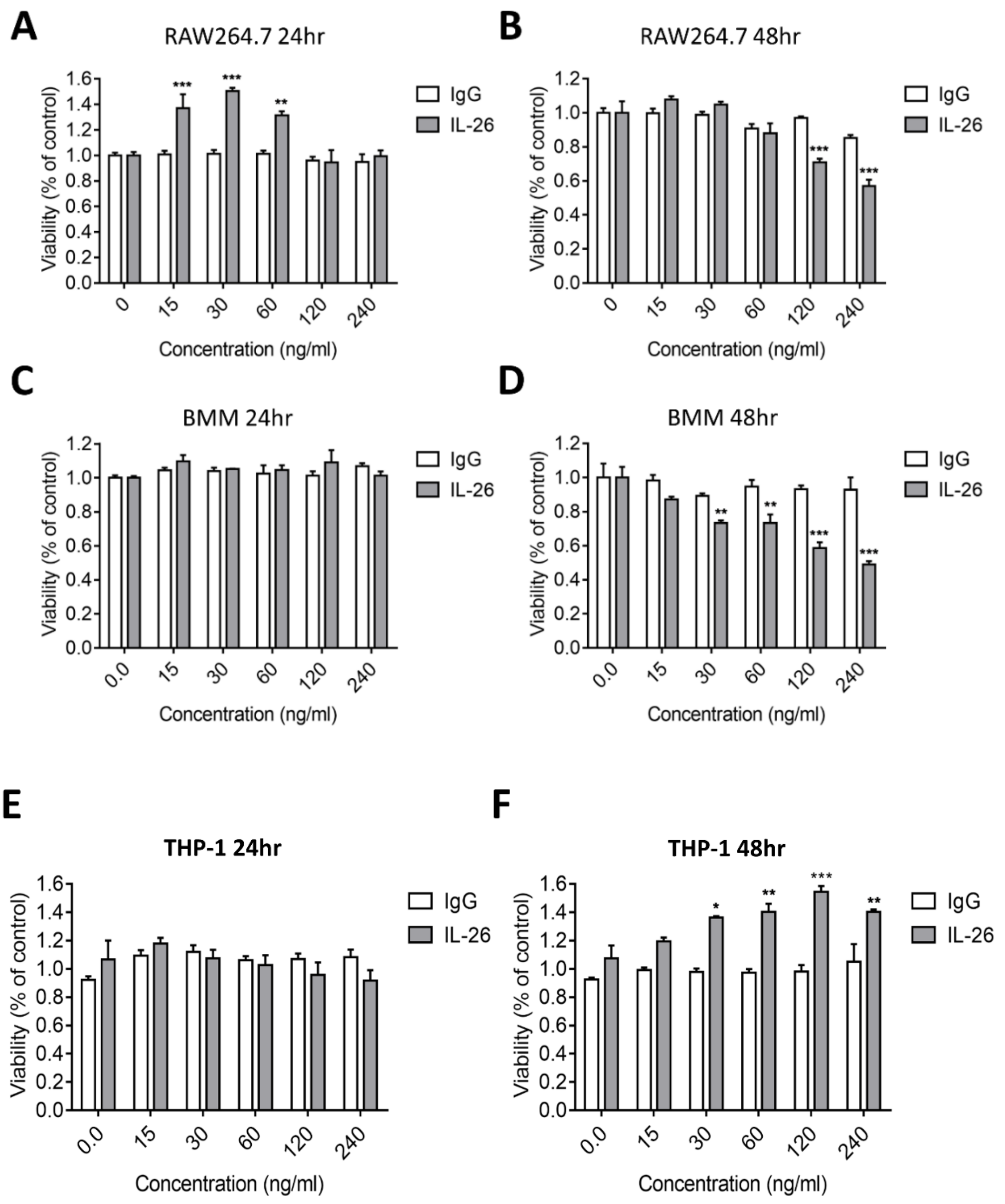

Figure S1. Effects of IL-26 on viability of RAW264.7, BMDM, and THP-1. (A, B) RAW264.7 cells, (C, D) Bone-marrow-derived macrophage (BMDM), or (E, F) THP-1 cells were seeded onto 96-well plates. Then the cells were cultured for 24 (A, C, E) or 48 (B, D, F) hours with the indicated concentrations of IL-26 or IgG control. After 24 or 48 hours, MTT solution (500 μg/ml) was added to each well for another an hour incubation. After incubation, the optical density was read at 570 nm in an ELISA plate reader. Cell viability was determined relative to the control. The data represent the means ± S.D. of more than 3 cultures. (**<0.05, **P<0.01, ***P<0.001)

Figure S2

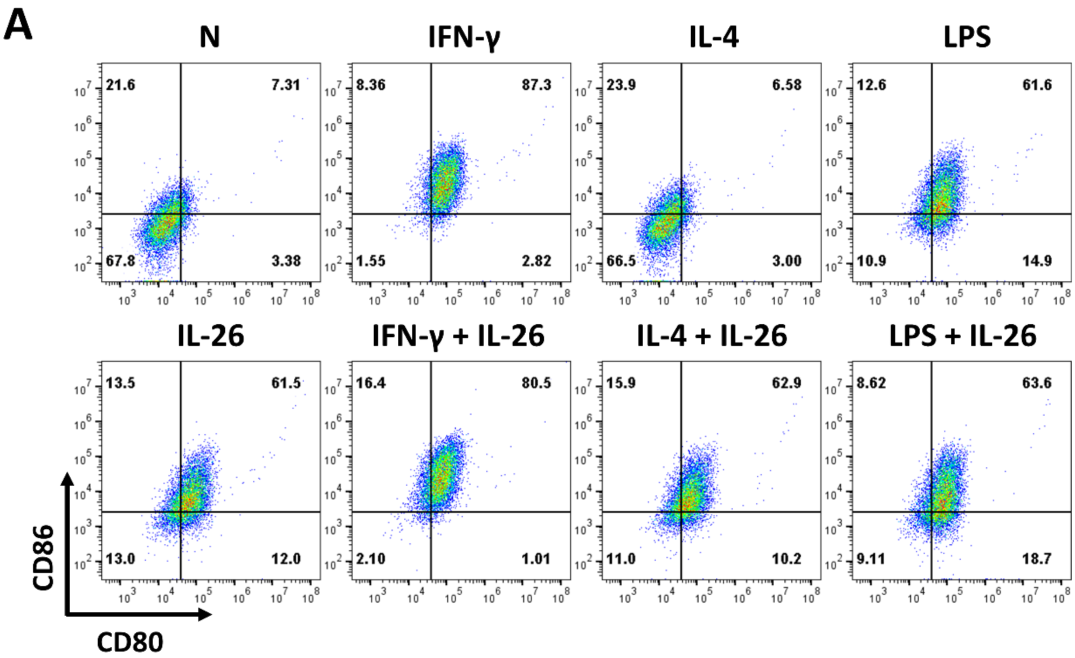
A
N
IFN-γ
IL-4
LPS
21.6
7.31
67.8
3.38
8.36
87.3
1.55
2.82
23.9
6.58
66.5
3.00
12.6
61.6
10.9
14.9
IL-26
IFN-γ + IL-26
IL-4 + IL-26
LPS + IL-26
13.5
61.5
13.0
12.0
16.4
80.5
2.10
1.01
15.9
62.9
11.0
10.2
8.62
63.6
9.11
18.7
CD86
CD80

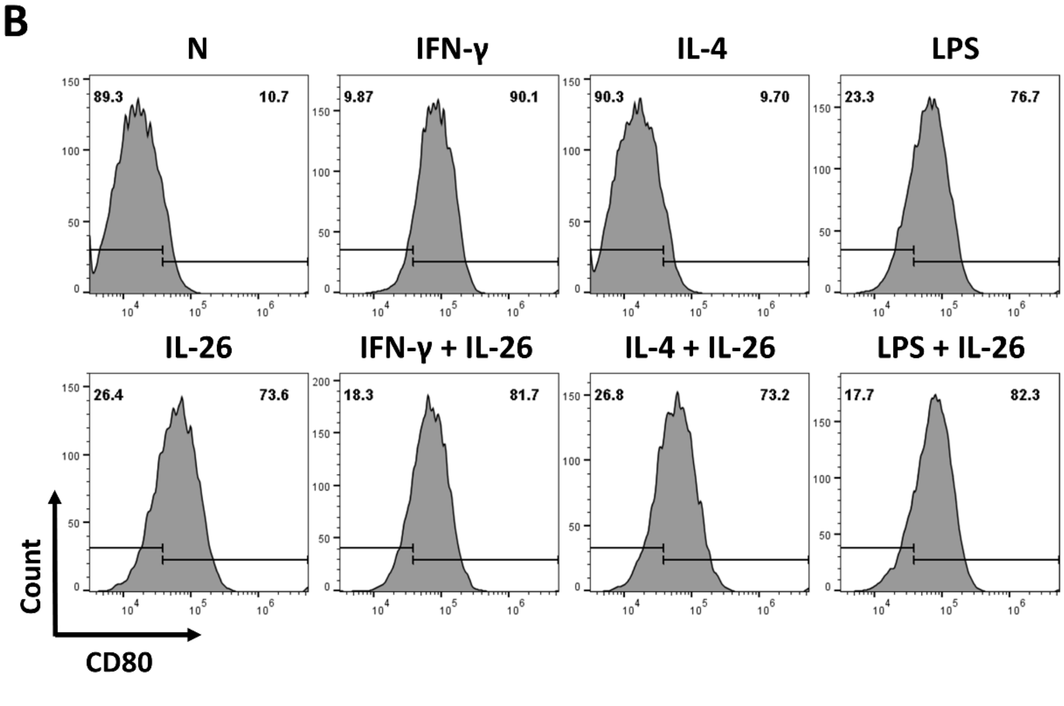
B
N
IFN-γ
IL-4
LPS
89.3
10.7
9.87
90.1
90.3
9.70
23.3
76.7
IL-26
IFN-γ + IL-26
IL-4 + IL-26
LPS + IL-26
26.4
73.6
18.3
81.7
26.8
73.2
17.7
82.3
Count
CD80

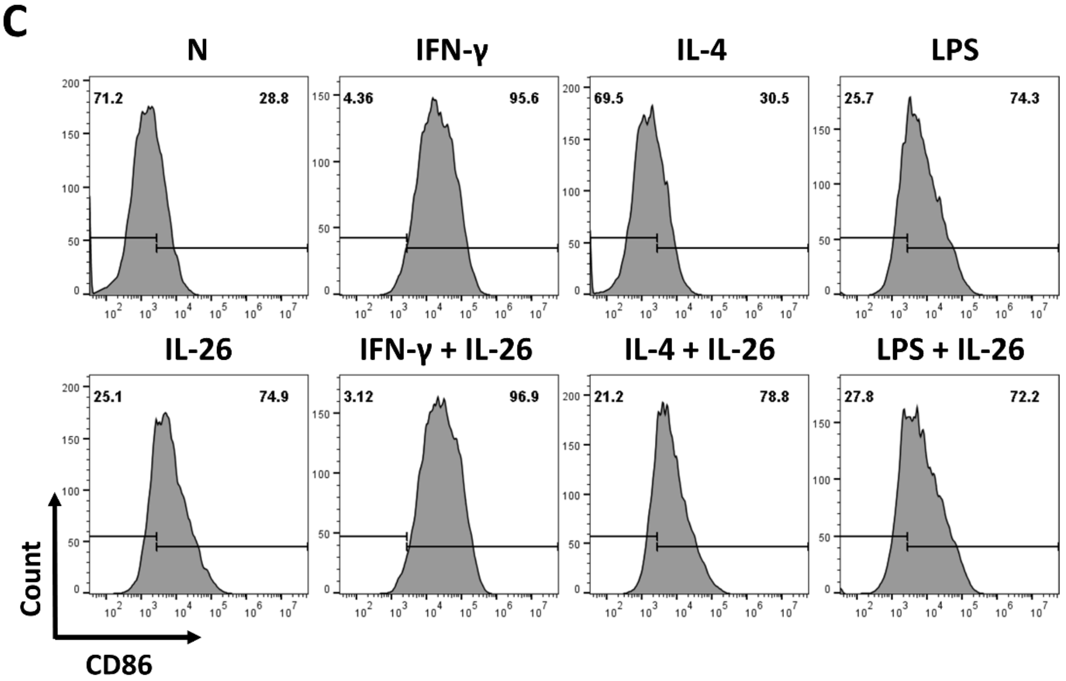

Figure S2. Effect of IL-26 on M1 CD80/CD86 macrophage marker expression in RAW264.7 (A-C) RAW264.7 cells were treated with IFN-γ (20 ng/ml), IL-4 (20 ng/ml), or LPS (10 ng/ml) in the presence or absence of IL-26 for 24 hours. After incubation, the cells were stained with M1 CD80 and CD86 marker to monitor (B) CD80 and (C) CD86 expression. A representative result of at least three independent experiments is shown.

Figure S3

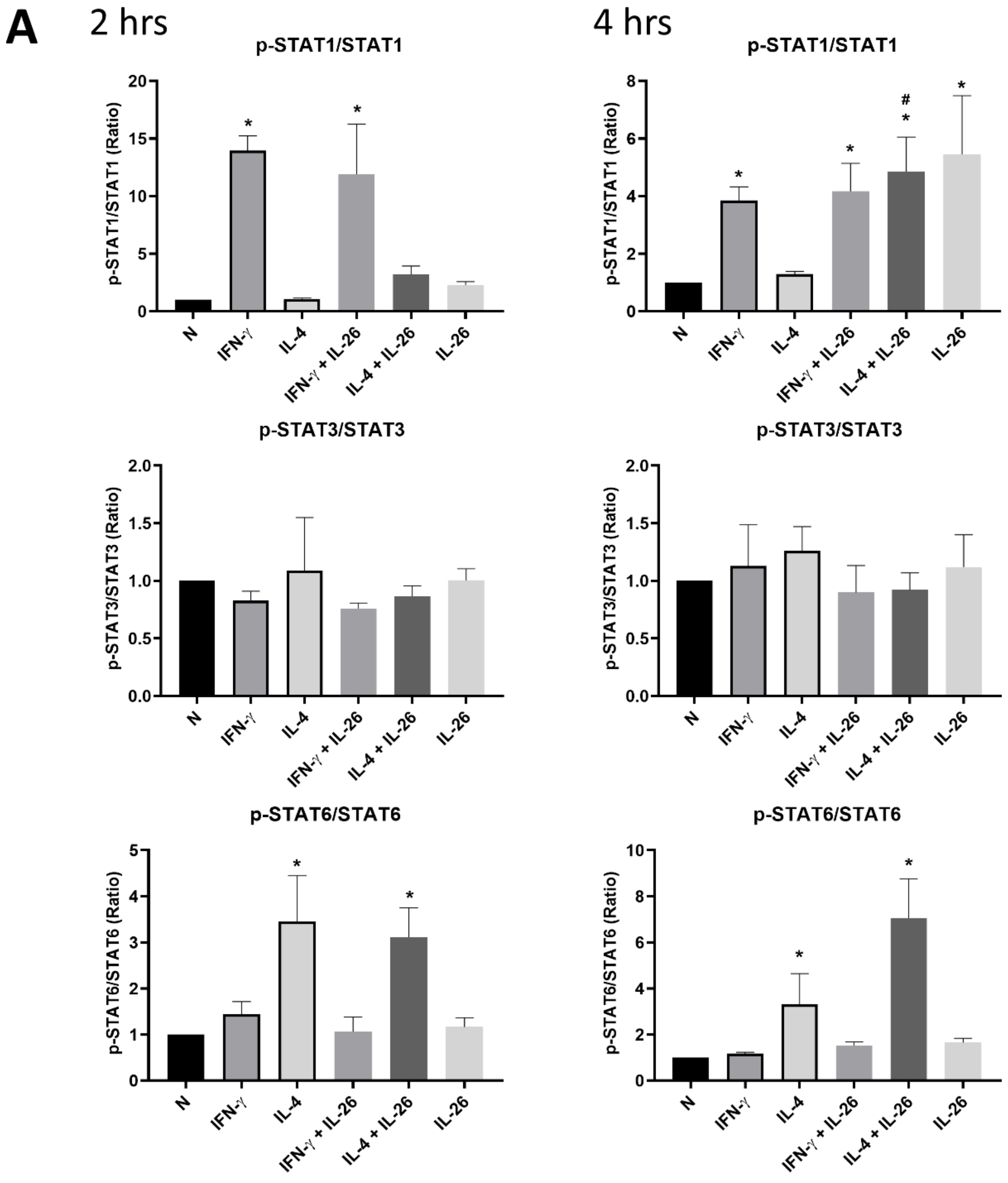
A
2 hrs
4 hrs
p-STAT1/STAT1
p-STAT1/STAT1 (Ratio)
p-STAT3/STAT3
p-STAT3/STAT3 (Ratio)
p-STAT6/STAT6
p-STAT6/STAT6 (Ratio)
N
IFN-γ
IL-4
IFN-γ + IL-26
IL-4 + IL-26
IL-26

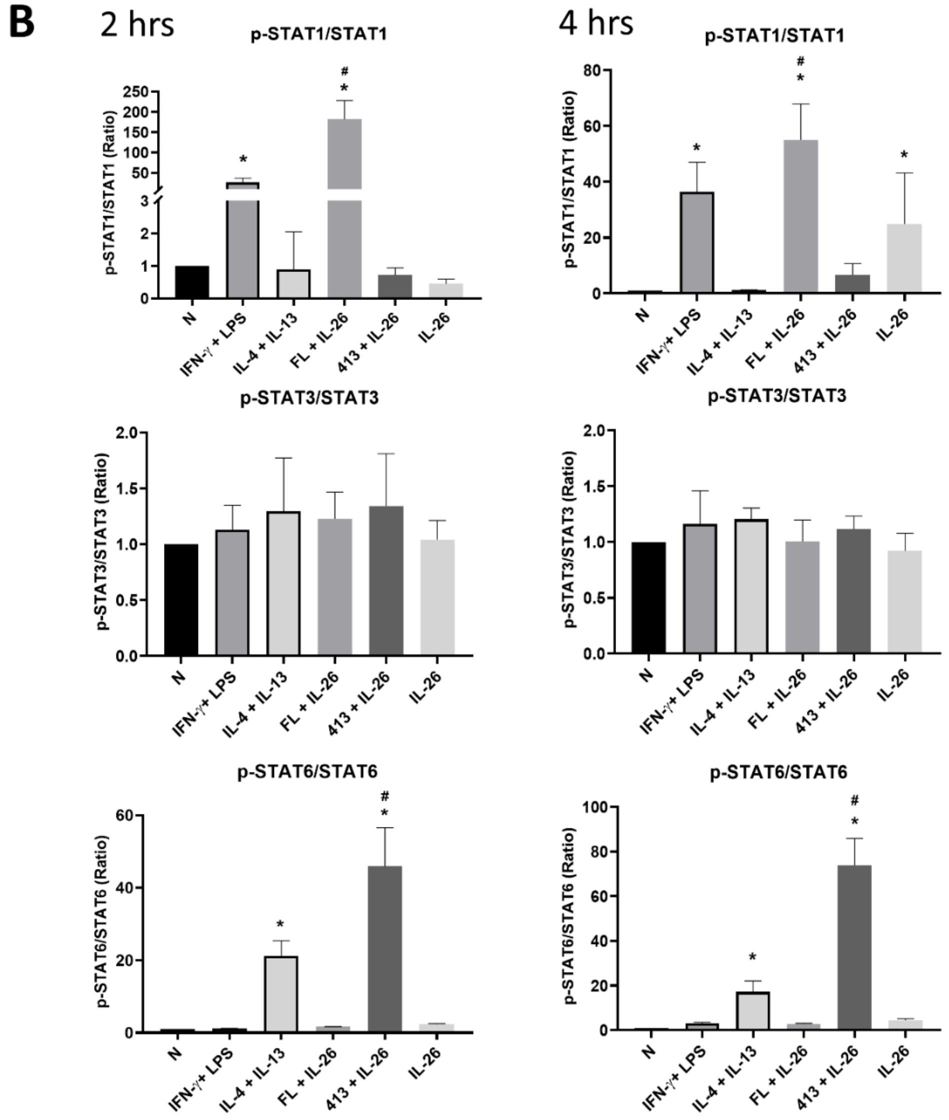

Figure S3. Effect of IL-26 on STAT1, STAT3, and STAT6 activation in M1/M2 macrophage differentiation. (A)RAW264.7 cells were serum-starved for 16 hours and treated with IFN-γ (20 ng/ml) or IL-4 (20 ng/ml) in the presence or absence of IL-26 and (B) THP-1 cells were treated with IFN-γ (20 ng/ml) plus LPS (10 ng/ml) or IL-4 (20 ng/ml) plus IL-13 (20 ng/ml) in the presence or absence of IL-26 for 2 and 4 hours to further detect phosphorylated or non-phosphorylated STAT1, STAT3, and STAT6 protein. Cell extracts were analyzed by western blot using antibodies specifically directed against the phosphorylated forms of STATs, compared with data obtained with antibodies directed against the unphosphorylated states of the kinases. Equal amounts of protein were loaded in each lane as demonstrated by the level of GAPDH. A representative result of at least three independent experiments is shown. (FL+IL-26: IFN-γ+LPS+IL-26; 413+IL-26: IL-4 + IL-13+IL-26; *P<0.05 comparison significance with N group; #P<0.05 multiple comparison significance between IL-4 and IL-4+IL-26, IL-4+IL-13 and IL-4+IL-13+IL-26, or IFN-γ+LPS and IFN-γ+LPS+IL-26).

Figure S4

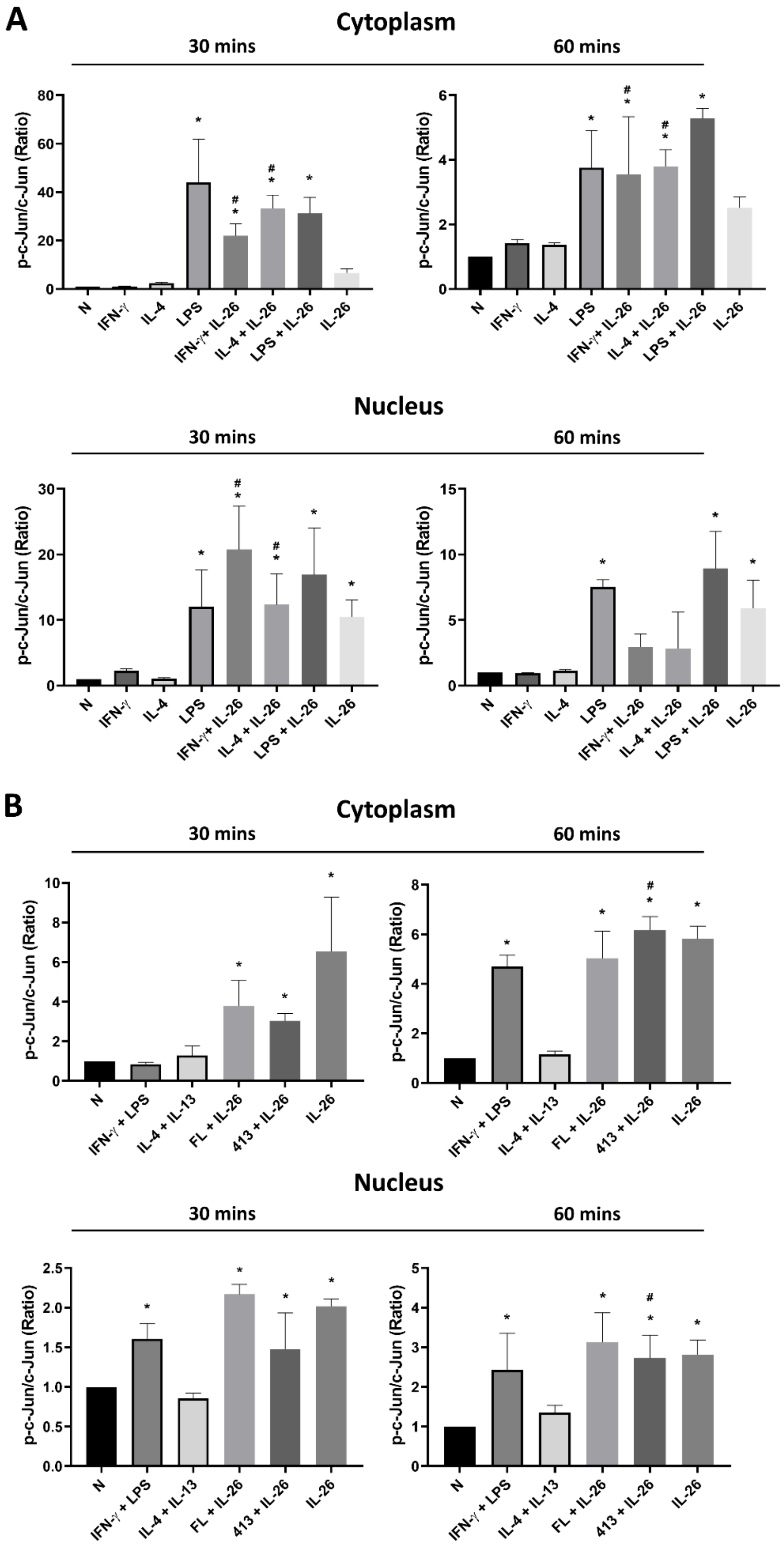
A
Cytoplasm
30 mins
60 mins
p-c-Jun/c-Jun (Ratio)
N
IFN-γ
IL-4
LPS
IFN-γ+ IL-26
IL-4 + IL-26
LPS + IL-26
IL-26
Nucleus
B
IFN-γ + LPS
IL-4 + IL-13
FL + IL-26
413 + IL-26

C

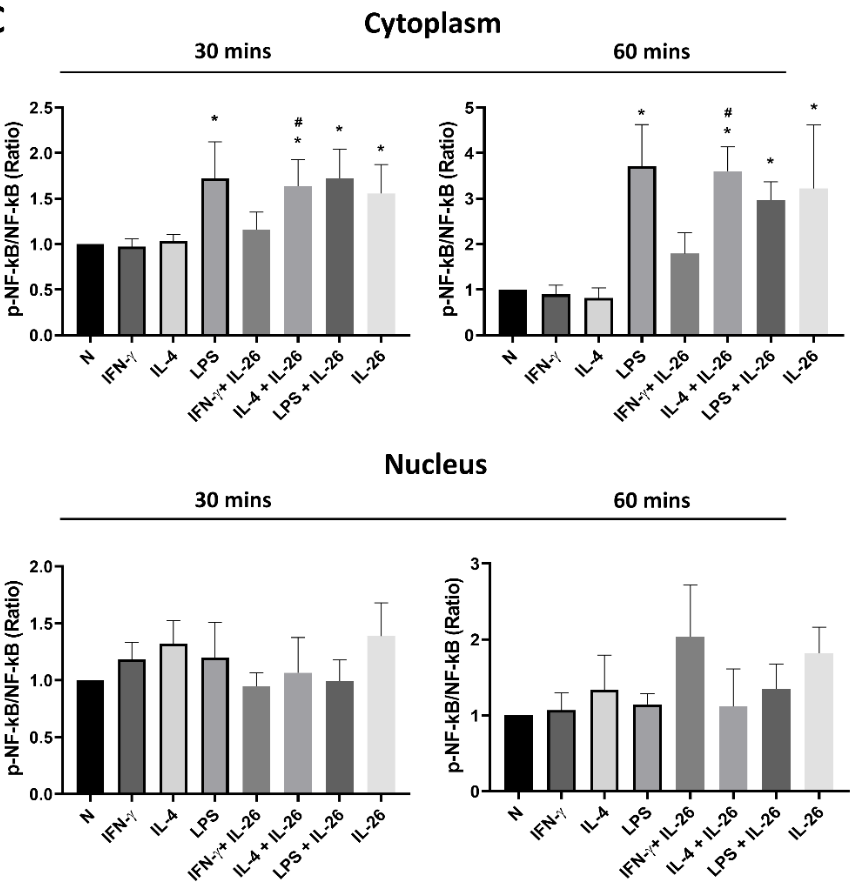

D

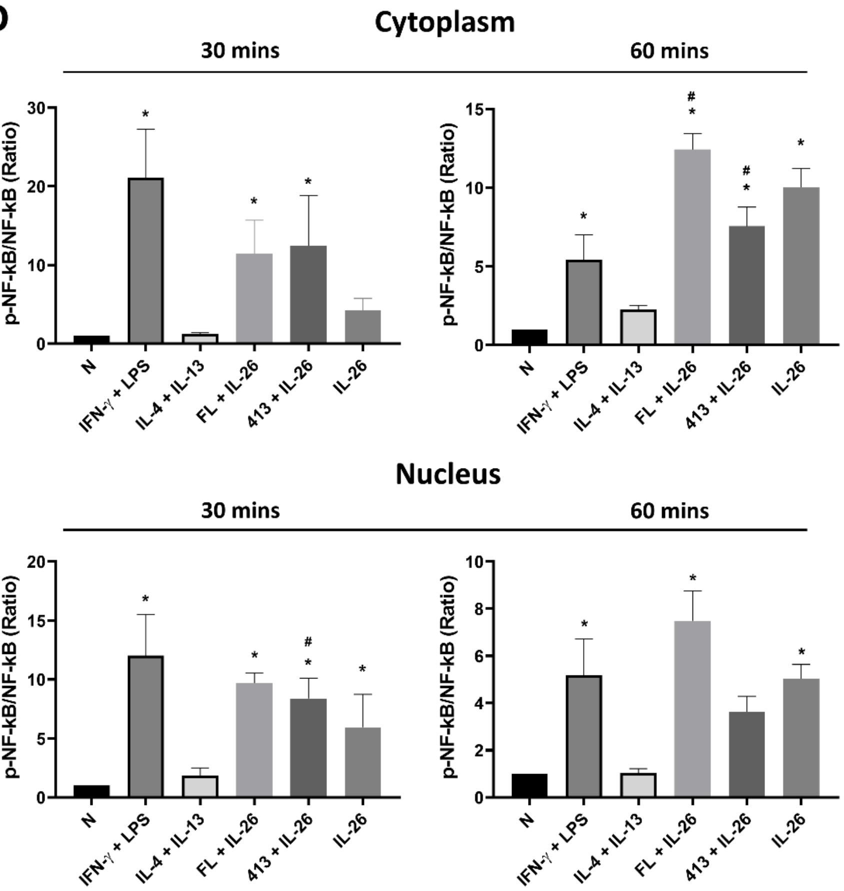

Figure S4. Effect of IL-26 on cJUN and NF-κB activation in M1/M2 macrophage differentiation. (A, C) RAW264.7 cells were serum-starved for 16 h and then treated with IFN-γ (20 ng/ml), IL-4 (20

ng/ml), or LPS (10 ng/ml) in the presence or absence of IL-26 for 30 or 60 mins. (B, D) THP-1 cells were treated with IFN-γ (20 ng/ml) plus LPS (10 ng/ml) or IL-4 (20 ng/ml) plus IL-13 (20 ng/ml) in the presence or absence of IL-26 for 30 or 60 mins. Cytoplasmic extracts (Cyto) and nuclear extracts (Nu) were analyzed by western blot using antibody specifically directed against phosphorylated or non-phosphorylated c-JUN (A, B) and NF-κB (C, D) protein. Equal amounts of protein were loaded in each lane as demonstrated by the level of TBP, nuclear internal control and GAPDH, cytoplasmic internal control. A representative result of at least three independent experiments is shown. (FL+IL-26: IFN-γ+LPS+IL-26; 413+IL-26: IL-4 + IL-13+IL-26; *P<0.05 comparison significance with N group; #P<0.05 multiple comparison significance between IL-4 and IL-4+IL-26, IL-4+IL-13 and IL-4+IL-13+IL-26, IFN-γ and IFN-γ+IL-26, or IFN-γ+LPS and IFN-γ+LPS+IL-26).

Figure S5

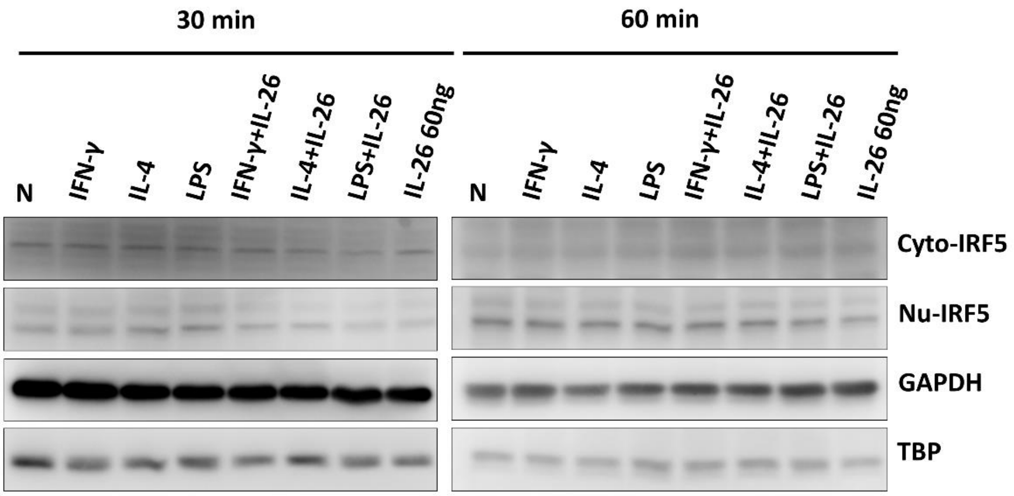

Figure S5. Effect of IL-26 on IRF5 translocation in M1/M2 macrophage differentiation. RAW264.7 cells were serum-starved for 16 h and then treated with IFN-γ (20 ng/ml), IL-4 (20 ng/ml), or LPS (10 ng/ml) in the presence or absence of IL-26 for 30 or 60 mins. Cytoplasmic extracts (Cyto) and nuclear extracts (Nu) were analyzed by western blot using antibody specifically directed against IRF5 protein. Equal amounts of protein were loaded in each lane as demonstrated by the level of TBP, nuclear internal control and GAPDH, cytoplasmic internal control. A representative result of at least three independent experiments is shown.

Figure S6

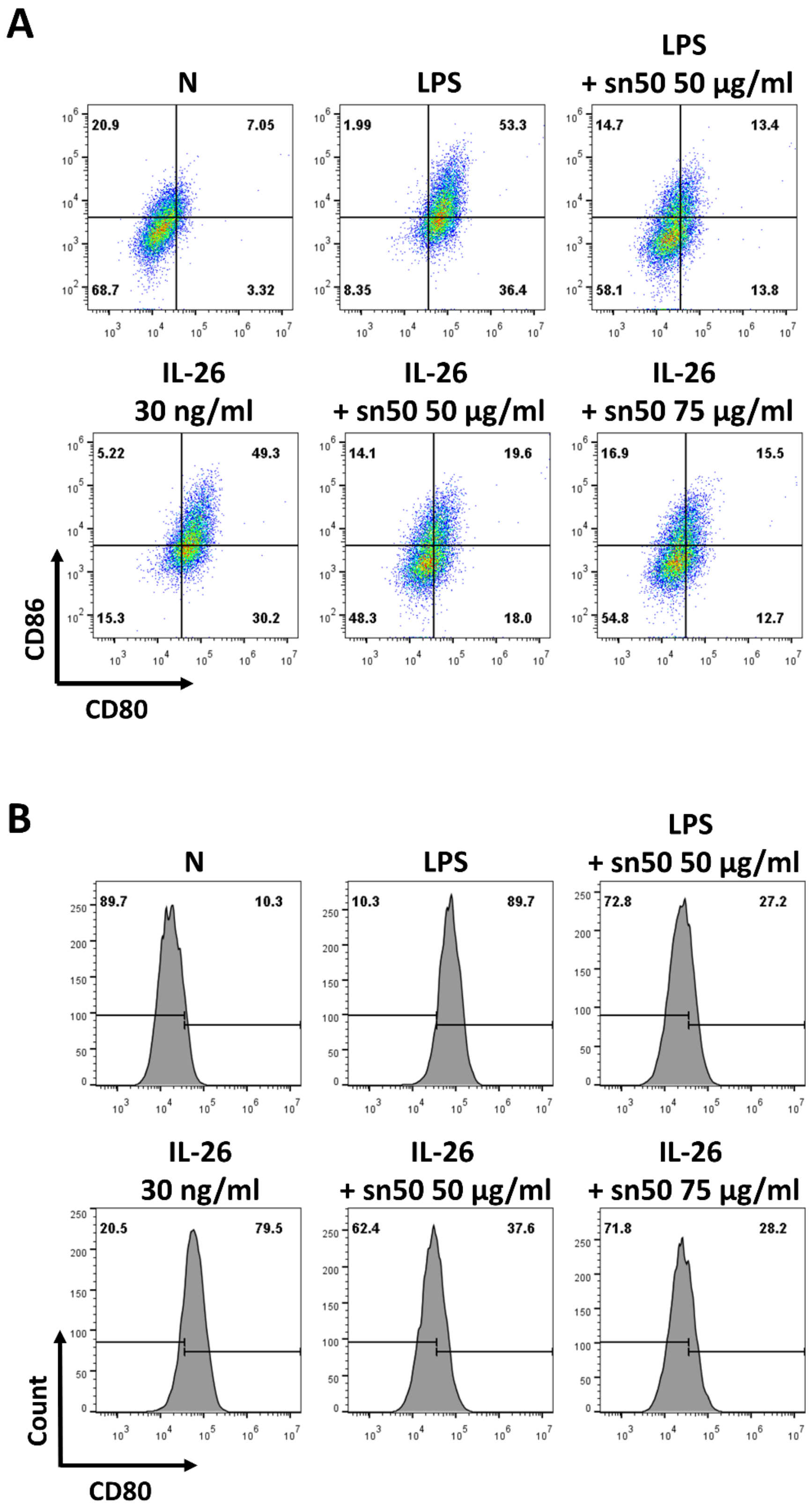

A
N
LPS
LPS
+ sn50 50 µg/ml
20.9
7.05
68.7
3.32
1.99
53.3
8.35
36.4
14.7
13.4
58.1
13.8
IL-26
30 ng/ml
IL-26
+ sn50 50 µg/ml
IL-26
+ sn50 75 µg/ml
5.22
49.3
15.3
30.2
14.1
19.6
48.3
18.0
16.9
15.5
54.8
12.7
CD86
CD80
B
N
LPS
LPS
+ sn50 50 µg/ml
89.7
10.3
10.3
89.7
72.8
27.2
IL-26
30 ng/ml
IL-26
+ sn50 50 µg/ml
IL-26
+ sn50 75 µg/ml
20.5
79.5
62.4
37.6
71.8
28.2
Count
CD80

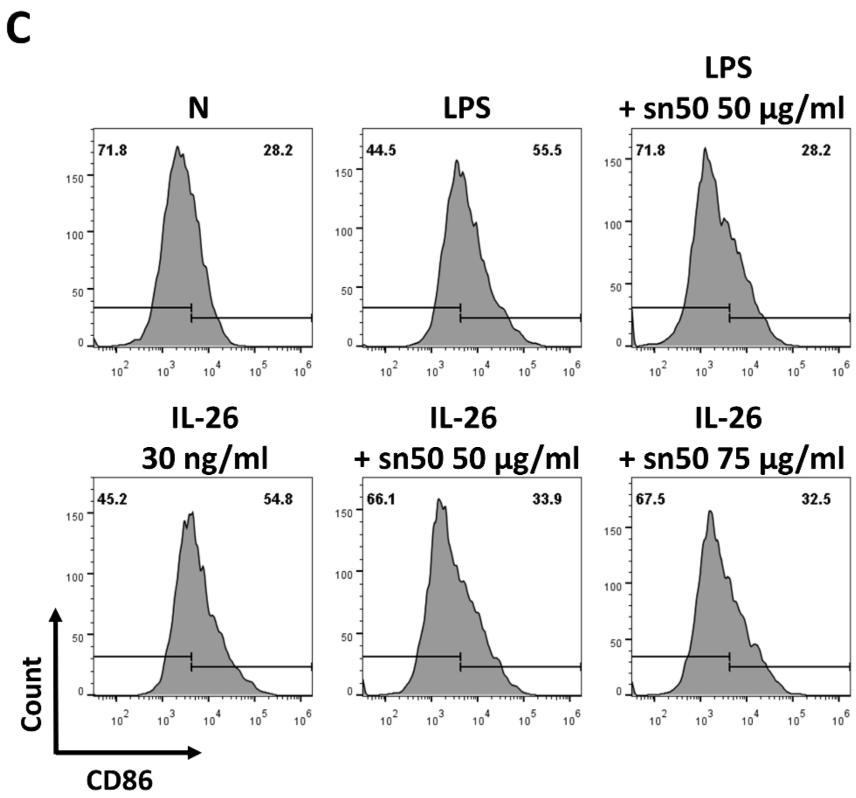

Figure S6. Suppression of IL-26 on STAT1, AP-1 and NF-κB activation by sn50 inhibitor. (A)RAW264.7 was pretreated with sn50 (50μg/mL or 75μg/mL) for 1 hour then treated with half dosage of LPS (5ng/ml) or IL-26 (30 ng/ml) for 16 hours. After 16 hours incubation, CD80 (B) and CD86 (C) surface marker were stained and analysis by flowcytometry. A representative result of at least three independent experiments is shown.

Supplementary Table

Table 1. List of murine PCR primers.

Genes	Nucleotide sequences
TNF-α	Forward: CCCTCACACTCACAAACCAC
	Reverse: GGCAGAGAGGAGGTTGACTT
iNOS	Forward: TCAGCTACGCCTTCAACACC
	Reverse: TTCCCAAATGTGCTTGTCACC
CD80	Forward: TTCGTCTTTCACAAGTGTCTTCA
	Reverse: TGCCAGTAGATTCGGTCTTCA
IL-10	Forward: GCTCTTACTGACTGGCATGAG
	Reverse: CGCAGCTCTAGGAGCATGTG
CD206	Forward: CATTCCCTCAGCAAGCGATG
	Reverse: GGGTTCCATCACTCCACTCA
